# Evaluation of the quality of CT-like images obtained using a commercial flat panel detector system

**DOI:** 10.2349/biij.2.4.e48

**Published:** 2006-10-01

**Authors:** JM Smyth, DG Sutton, JG Houston

**Affiliations:** 1 Department of Medical Physics, Ninewells Hospital and Medical School, Dundee, Scotland; 2 Department of Radiology, Ninewells Hospital and Medical School, Dundee, Scotland

**Keywords:** CT-like images, flat panel detector, image quality

## Abstract

**Purpose:**

The development of flat panel detector technology has resulted in renewed interest in the possibility of generating CT-like images from rotational angiographic acquisitions. At least two commercial products now use cone beam reconstruction software in conjunction with flat panel detectors to produce such images. The purpose of the work presented here is to report on image quality obtained from one such system in objective and subjective terms and to compare it with the quality of images obtained from a modern multi-detector CT scanner.

**Method:**

The Image quality was assessed using a CATPHAN 500 model and an AAPM CT Performance Phantom model. Image noise, CT number accuracy, CT number consistency, Low Contrast Resolution, surface dose and Modulation Transfer Function were assessed for the flat panel detector and compared with results obtained from a 4 slice CT scanner.

**Results:**

As expected image quality obtained from the CT scanner was much better than from the flat panel detector. Low contrast resolution was much worse and the surface dose was higher for the flat panel detector than the CT scanner. There was an inaccuracy in CT number determination and the noise was greater by a factor of two or three. Limiting resolution was better on images from the CT scanner.

**Conclusion:**

The poor low contrast resolution from flat panel detector was expected given the expected resolution of ±10 Hounsfield Units. These systems should not be considered as diagnostic CT scanners. However, the remaining performance figures indicate that the CT-like images obtained from this type of equipment are of sufficient quality for at least some clinical applications, such as detection of brain haemorrhages in the vascular suite.

## INTRODUCTION

**Figure 1:**
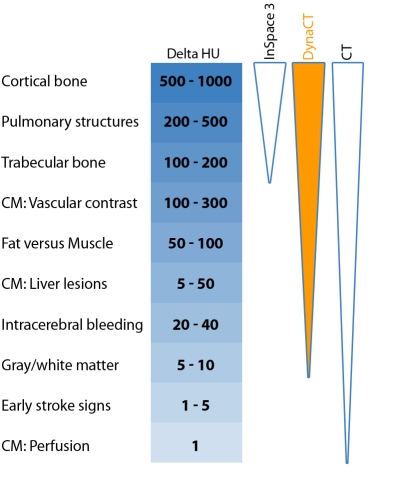
Hounsfield unit resolution (Delta HU) required for a range of clinical indications with arrows showing minimum contrast resolution for rotational angiography, DynaCT and conventional CT.

Rotational angiography is an image acquisition technique which was designed to overcome some of the limitations of traditional angiography introduced by the two dimensional depiction of complex three dimensional structures. A major driving force behind the development of rotational angiography has been the ability to apply CT-like algorithms for 3D volumetric reconstructions [1]. A consequence of the rendering approach is that the resulting images lack the contrast resolution of true CT, being typically able to resolve differences of some 100 Hounsfield Units (HU) compared to 1 HU for conventional CT (Figure 1). Active matrix flat panel detectors have recently been developed for fluoroscopic imaging [2] and are increasingly being incorporated into angiographic equipment. Such detectors provide the efficient, practically distortion free environment that is required for cone beam CT reconstruction [3]. Consequently, the development of flat panel detector technology has resulted in renewed interest in the possibility of generating CT-like images from rotational angiographic acquisitions. The potential increase in contrast resolution to almost 10 HU may have considerable implications during angiographic procedures. For example, in neuroradiology, brain haemorrhages resulting from pathology or during a coiling process may be visualised, and in abdominal radiology it may be possible to image vessels without the introduction of a contrast agent (Figure 1). At least two major manufacturers have products that utilise cone beam reconstruction software in conjunction with flat panel detector to produce CT-like images from rotational angiography acquisitions. There is very little published data on the utility of these systems; one paper discusses the utility of CT-like images in neuroendovacular procedures [4] and reports that the quality of the images is sufficient for diagnosis.

The ability to use axial 2D in an interventional radiology suite rather than a CT scanner room has distinct advantages in patient monitoring, access for procedural instruments and devices, supportive equipment and staff familiarisation. Such advantages may offset the poorer image quality achieved, particularly if a combination of CT-like images and standard 2D and 2D rotational techniques are employed. However, a better understanding of the relative image quality differences between the CT-like images obtained using rotating flat panel detector systems and those obtained in the CT suite is essential for further progress in this area. The purpose of the work presented here is to report on the image quality obtained from one such system in both objective and subjective terms and to compare it with the quality of images obtained from a modern multi-detector CT scanner.

## HARDWARE

**Figure 2:**
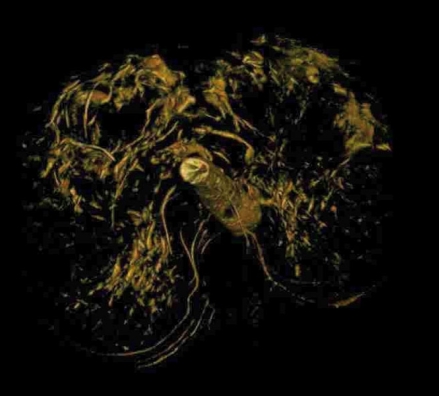
Example of renal subtraction images obtained using 3D rotational angiography.

**Figure 3:**
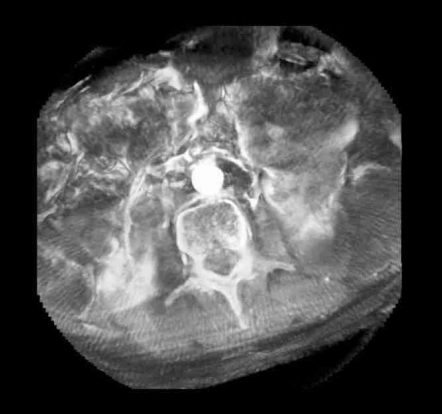
The same volume as shown in Figure 2 but processed using DynaCT.

The equipment used in this study, the Siemens Axiom Artis dTA, is a ceiling mounted C-arm angiography system with an amorphous silicon flat panel detector for use in interventional and diagnostic applications. In addition to digital fluoroscopy, subtraction and non subtraction digital acquisition, Axiom Artis dTA can perform rotational angiography (Dynavision) with 3D reconstructions displayed on an imaging workstation (Figure 2). Siemens Medical have developed specialised software (DynaCT) to allow reconstruction of CT-like data sets with soft tissue resolution from rotational angiographic acquisitions (Figure 3).

Images are acquired using a 30 cm x 38 cm amorphous silicon detector with 2480 x 1920 matrix size (154 μm pixel pitch) and 14-bit depth. The acquisitions are carried out using a partial (200°) rotation with the X-Ray tube moving underneath the table top. There are three rotation times (5s, 10s and 20s), which correspond to 133, 248 and 495 projection images at 1.5°, 0.8° and 0.4° increments. The kV and mA are selected using Automatic Exposure Control and depend on the detector dose selected: 0.36 μGy or 1.2 μGy. The X-Ray radiation is pulsed; typical pulse widths are 7.5ms or 11ms. The largest field size is the default although some collimation is theoretically possible. The stated contrast resolution is of the order of 10 HU. The source to detector distance is fixed at 118cm, giving a 25cm field of view at centre of rotation in the default configuration. This should be compared to a conventional CT scanner with scan fields of view up to 50cm at the isocentre for body acquisitions.

To acquire volumetric data from the cone beam projections, a modified Feldkamp reconstruction algorithm is utilised. The recommended reconstruction kernel is based on a Shepp-Logan filter but there is some suppression of high spatial frequencies and summation of several detector lines to reduce noise and increase low contrast resolution. Other corrections are applied to improve the images, such as measures to reduce the contribution from scattered radiation (software scatter correction), application of compensation for objects that do not fully fit into detector (truncation correction) and balancing of pixel response to correct for ring correction artefacts. Grey scale values are adjusted to an HU scale after the corrections have been applied.

The images are displayed in 256 x 256 matrix with 8 bit depth.

The multi-detector CT scanner used for comparison in this study is a GE LightSpeed Plus 4 slice scanner. An evaluation report for this scanner, including a technical summary, can be found on the UK NHS Purchasing and Supplies Agency (PASA) website [6].

## MATERIALS AND METHODS

**Figure 4:**
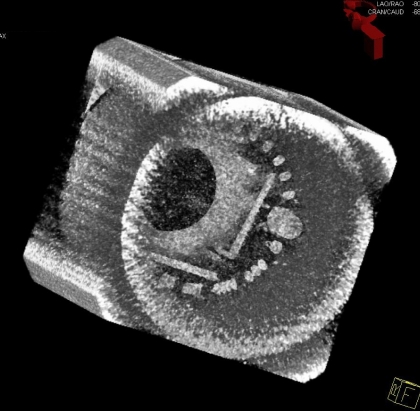
A CATPHAN 3D reconstruction processed using DynaCT.

Image quality was assessed using a CATPHAN 500 model (The Phantom Laboratory, Salem, NY) and an AAPM CT Performance Phantom model 76-410-4130 (Nuclear Associates, Carle Place, NY). Both these phantoms have diameters of 20cm and are similar in size to head phantoms (typically 16cm). Using larger, body sized phantoms would result in loss of peripheral detail, as the field of view is only 25cm at the centre of rotation (body phantoms typically have a 32cm diameter). Figure 4 shows a 3D reconstruction of the CATPHAN generated following a rotational acquisition.

The kV automatically selected by the AEC system was approximately 70 kV for both phantoms. The mAs automatically selected by the AEC system varied depending on the region of phantom being imaged and nominal dose to the detector. It is recognised that the kV and mAs values clinically used for head applications may vary to some extent from those selected for phantoms.

Noise analysis and CT number evaluation for flat panel detector images were performed on a Siemens Leonardo workstation using the software tools provided. MTF measurements were performed using an IDL program previously developed in-house for the analysis of CT images of a wire. DynaCT acquisitions were obtained with phantoms positioned on the table top and offset in the longitudinal direction so that centre of rotation coincided with centre of the imaging module in question.

The same phantoms were used to assess image quality on the CT scanner. An equivalent, or where possible, the same acquisition and image parameters were used similar to the flat panel detector system. Images were acquired in axial mode with 25cm field of view at the isocentre using a head bow tie filter, and displayed at 25cm field of view. All images were reconstructed using a 512 x 512 matrix and, unless otherwise stated, standard GE algorithm.

The Axiom Artis dTA system had undergone extensive acceptance and commissioning tests within six months of this study. An in-air calibration was performed on the CT scanner prior to acquisition of the images and routine image quality analysis is performed on a weekly basis.

### I. Image Noise and CT Number of Water

Image noise and CT number of water were determined from images of the CATPHAN CTP422 liquid bath module. Acquisitions were obtained at low dose (0.36 μGy/frame) with 5s and 10s rotations and at high dose (1.20 μGy/frame) with a 20s rotation time. The kV and mA were under AEC control and the field size was 30cm x 38cm at the flat panel detector (magnification zero).

**Figure 5:**
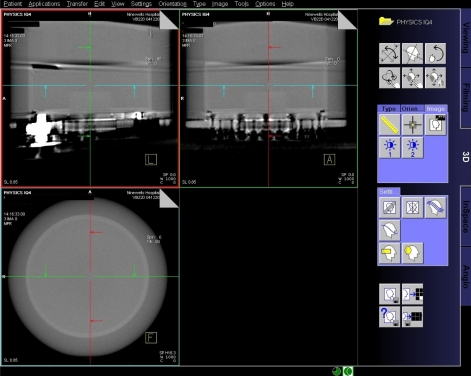
DynaCT volume data displayed as MPR images on Leonardo Workstation 3D card.

**Figure 6:**
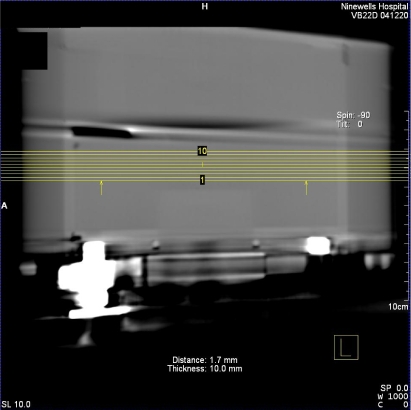
MPR tool used to create images at specific slice widths from a coronal view.

**Figure 7:**
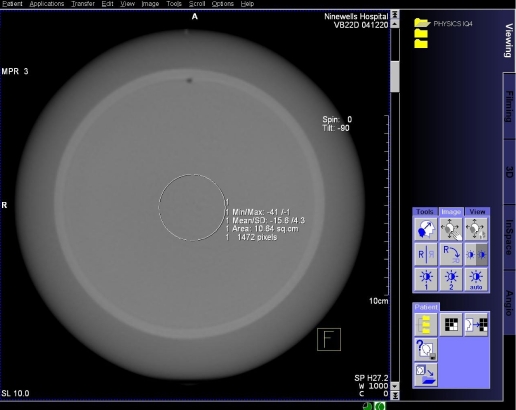
Axial image of CATPHAN Water module with ROI measurement tool.

Raw data was processed at the workstation using the default DynaCT InSpace reconstruction presets (Table 1). The volume data was displayed on the workstation 3D card (Figure 5) and a multiplaner formatting (MPR) tool was used to create axial images at specified image slice widths (Figure 6), as in conventional CT. Axial images were analysed on the workstation using the supplied measurement tools; a circular region of interest (approximately 1500 pixels) was drawn in centre of the image (Figure 7) and the area, mean pixel value and standard deviation (in pixel value) for the region of interest (σ_ROI_) were displayed. The CT number of water was taken to be the mean pixel value and the noise was as defined below.

$$\text{Percentage Noise = }\frac{\sigma_{ROI}x100}{CT_{H_{2}O_{}}-CT_{air}}$$

where CT_H2O_ and CT_air_ are the CT numbers of water and air respectively. A single image in centre of phantom was analysed for each of the four acquisitions.

**Table 1:** DynaCT InSpace reconstruction presets

**Parameter**	**Setting**
Volume of Interest	Full
Slice Matrix	256 x 256
Kernel	Bone
Image Characteristics	Smooth
Reconstruction	Subtraction
Viewing Preference	DynaCT Soft Tissue 1_2005
Ring Correction	Yes
Scatter Correction	Yes
Truncation Correction	Yes

For conventional CT, the centre of the CATPHAN CTP422 liquid bath module was placed at the scanner isocentre. Axial images were acquired and analysed on the CT scanner console using the supplied measurement tools; a circular region of interest (approximately same physical size) was drawn in centre of the area, mean pixel value and standard deviation (in pixel value) for the region of interest (σ_ROI_) were displayed. The CT number of water was taken to be the mean pixel value and the noise was as defined as above.

### II. CT Number Consistency

To check CT consistency, a CATPHAN CTP401 sensitometry module containing four materials of differing attenuation: air (75% N, 23.2% O, 1.3% Ar), LDPE (C_2_H_4_), Teflon (CF_2_) and acrylic (C_5_H_8_O_2_), each of 1.2cm in diameter was positioned symmetrically at 1.8cm from edge of the phantom. Acquisitions were obtained at the low dose setting (0.36 μGy/frame) with 5s rotation time. The kV and mA were under AEC control and the field size was 30cm x 38 cm at the flat panel detector.

**Figure 8:**
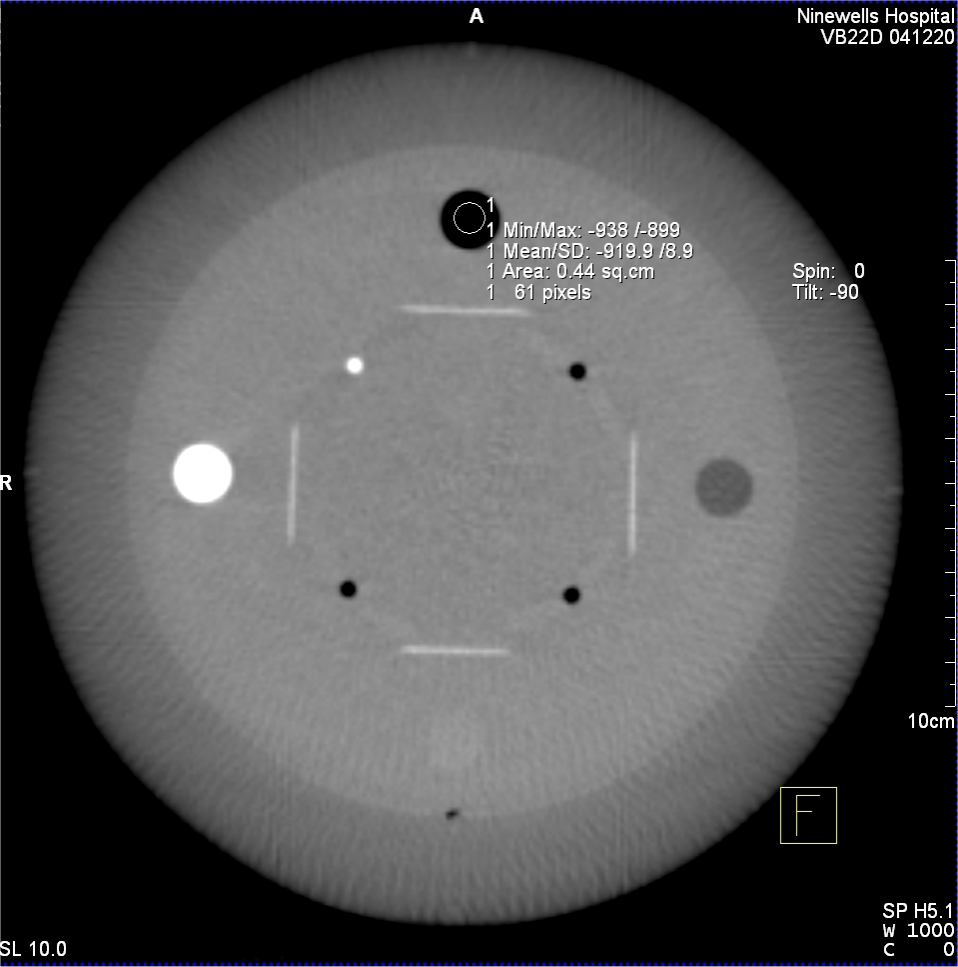
Axial image of CATPHAN sensitometry module with ROI measurement tool. Note the radial streaks characteristic of photon starvation.

Axial images were generated, as described in the previous section, and analysed using the supplied measurement tools. A circular region of interest was drawn in centre of each material (Figure 8) and the area, mean pixel value and standard deviation (in pixel value) for region of interest (σ_ROI_) were generated. The CT number of each material was taken to be the mean pixel value. The final result was obtained by averaging the data obtained from three adjacent axial images.

For conventional CT, the centre of CATPHAN CTP401 sensitometry module was placed at the scanner isocentre. Axial images were acquired and analysed on the CT scanner console using the supplied measurement tools. A circular region of interest was drawn in centre of each material and the area, mean pixel value and standard deviation (in pixel value) for region of interest (σ_ROI_) were generated. The CT number of each material was taken to be the mean pixel value.

### III. Low Contrast Resolution

Low contrast resolution was assessed using a CATPHAN CTP515 low contrast module containing low contrast target discs arranged in three groups with nominal contrast of 0.3%, 0.5% and 1.0% and decreasing diameters of 15mm, 9mm, 8mm, 7mm, 6mm, 5mm, 4mm, 3mm and 2mm. The acquisitions were taken at low dose (0.36 μGy/frame) with 10s and 20s rotations and at high dose (1.20 μGy/frame) using a 20s rotation time. The kV and mA were under AEC control and the field size was 30 cm x 38 cm at the flat panel detector. The dose was measured on the anterior surface of the phantom using a pencil ionisation chamber (Vertec PC-4P CT chamber).

**Figure 9:**
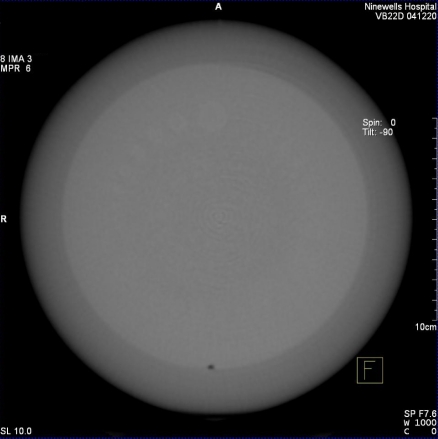
Axial image of CATPHAN LCD module generated using DynaCT.

Axial images were generated and displayed on the workstation using the default window levels (Figure 9). The number of discernable discs in each nominal contrast group were recorded by a single observer and corresponding disc diameter identified.

For conventional CT, the centre of CATPHAN CTP515 low contrast module was placed at the scanner isocentre. The dose was measured on the anterior surface of the phantom using the same pencil ionisation chamber. Axial images were displayed on the CT scanner console using default window levels. The number of discernable discs in each nominal contrast group were recorded by a single observer and corresponding disc diameter identified.

### IV. Modulation Transfer Function (MTF)

MTF was determined using an AAPM CT Performance Phantom model 76-410-4130 containing a stainless steel wire of 0.355 mm diameter and 15 cm length positioned 5.0 cm off centre in longitudinal (z-axis) direction. The acquisitions were obtained at low dose (0.36 μGy/frame) using a 5 s rotation time. As before, the kV and mA were under AEC control and field size 30 cm x 38 cm at the detector surface. The axial images were generated and transferred in DICOM format to a remote computer for analysis using in-house software.

The software uses an estimated point spread function obtained from radially averaging a series of pixel value profiles across the image of the wire. The MTF is derived from the modulus of the Fourier Transform of this function and is expressed as the frequency in cycles per cm corresponding to 50% and 10% modulation.

In the case of conventional CT, the AAPM CT Performance Phantom was positioned off centre so that the wire was within 1cm of the scanner isocentre. Axial images were generated and transferred in DICOM format to a remote computer for analysis using the in-house software described above.

## RESULTS & DISCUSSION

### I. Image Noise and CT Number of Water

The results are presented in Table 2 which also shows the CT number and noise obtained from a GE LightSpeed Plus CT scanner using a) generic exposure parameters at 80kV and b) standard brain protocol of 120kV, 240 mAs and a 10mm slice width on the same phantom.

**Table 2:** Image noise and CT number of water using standard reconstruction parameters

**Dose (μGy/frame)**	**Rotation Time (s)**	**kV**	**Average mA**	**Image Slice Thickness (mm)**	**CT Number**	**% Noise**	**ROI cm^2^**
0.36	5	70	264	10	-8	1.2	4.92
0.36	10	70	255	10	-22	1.0	4.92
0.36	10	70	255	2	-21	1.4	4.93
1.20	10	70	450	10	-22	0.48	4.97
CT 80	1	80	200	5	0	0.8	5.00
CT Brain	2	120	120	10	0	0.2	5.00

In conventional CT, the system is calibrated to give a CT number of zero for water, which was the value obtained for both sets of scanning parameters. The CT number from the DynaCT acquisition varied from -8 to -22. This must be interpreted within the context that the quoted resolution is +/- 10 HU and given that the modality is designed to be used in an interventional suite, cannot be considered to represent a significant error.

Theoretically, the image noise depends on the number of photons and varies with the reciprocal of the square root of mA, time and image slice width:

Noise^2^ ∝ (mAs * slice width)^-1^

This exact relationship may not hold if additional corrections are applied in processing but noise will increase if mAs or slice width are reduced, as observed in Table 2 for both DynaCT and conventional CT. The noise is greater in DynaCT in all but the highest dose option, as might be expected given the difference in the number of projections used for image reconstruction. Nevertheless, at between 1-1.5% noise values are fit for this purpose.

### II. CT Number Consistency

Three adjacent images were analysed and the average CT number obtained for each of the test materials; Air, LDPE, Acryllic and Teflon is presented in Table 3. In addition, results are given for a GE LightSpeed Plus CT (4 slice) scanner using the same phantom with similar exposure parameters (80 kV) and a more conventional potential of 120 kV.

**Table 3:** CT number consistency using standard reconstruction parameters

**Dose Level (μGy/frame)**	**Rotation Time (s)**	**kV**	**Average mA**	**Image Slice Thickness (mm)**	**CT Number Air**	**CT Number LDPE**	**CT Number Acrylic**	**CT Number Teflon**	**ROI cm^2^**
0.36	5	70	215	10	-911	-151	81	945	0.4
CT 80	1	80	200	5	-1006	-127	101	1010	0.4
CT Brain	1	120	200	5	-1001	-96	121	956	0.4

**Figure 10:**
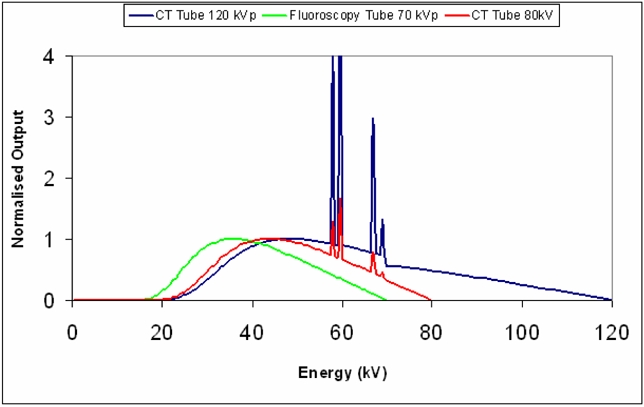
Comparison of CT tube spectra to fluoroscopic tube spectra. Fluoroscopic Spectrum 3mm Al, 70 kV. CT spectra 7 mm Al, 80 kV and 120 kV. The figure shows the difference in quality between the three spectra resulting from the difference in filtration and accelerating potential. Each spectrum is normalised to its own maximum.

Although there is no *de facto* right answer for the CT number of the test materials (with the exception of water) the DynaCT ‘CT number’ for LDPE, Acryllic and Teflon is lower than the number obtained from the CT scanner. Inspection of Table 2 shows the same is true in the case of water. The DynaCT ‘CT’ number for air is higher than that obtained from the CT scanner at either kV. The CT number is related to linear attenuation coefficient, which is a function of energy. The CT number of a material is therefore not a constant and will depend on the incident X-Ray spectrum, which in turn depends on the tube characteristics such as filtration and potential. It is not unexpected, therefore that values for the materials are not the same as for conventional CT scanner given the differences in CT X-Ray spectra compared to fluoroscopy X-Ray spectra (Figure 10) [5] which is considerably “softer” because of the reduced filtration.

It is worth noting that the DynaCT image of Figure 8 shows the radial streaking characteristic of photon starvation probably caused by combination of high attenuation materials in the module and minimal number of projections (133) obtained.

### III. Low Contrast Resolution

The results are shown in Table 4 which also shows the low contrast resolution obtained from a GE LightSpeed Plus CT scanner using the standard brain protocol of 120 kV, 240 mAs and a 10mm slice width on the same phantom. The results from the DynaCT system are, as one would expect, demonstrably inferior. For example, the standard CT brain protocol at 120 kV is capable of identifying a 5mm disc at 0.3% contrast with a surface dose of 6 mGy. The DynaCT system only manages to identify the 5mm disc at a contrast of 1% and requires a surface dose of at least 17 mGy. Increasing the dose does not improve the low contrast resolution noticeably and the lowest dose DYNA option does not even identify a 15mm disc at 1% contrast.

**Table 4:** Low contrast resolution using standard reconstruction parameters

**Dose Level (μGy/frame)**	**Rotation Time (s)**	**kV**	**Average mA**	**Image Slice Thickness (mm)**	**Diameter of disc at 0.3%**	**Diameter of Disc at 0.5%**	**Diameter of Disc at 1.0%**	**Anterior Surface Dose (mGy)**
0.36	10	70	243	10	not visible	not visible	not visible	9
0.36	20	70	235	10	15 mm	9 mm	5 mm	17
1.20	20	71	454	10	15 mm	8 mm	5 mm	52
CT 120	1	120	200	10	5 mm	3 mm	2 mm	6

### IV. Modulation Transfer Function

The results for MTF50% and MTF10% (line pairs/cm) are presented in Table 5 which also shows typical MTF values for two algorithms used in the reconstruction of images of the same phantom obtained with a GE LightSpeed Plus CT scanner. As can be seen, the MTF for conventional CT is slightly better for standard algorithms and considerably better for high resolution edge enhancement algorithms. The 10% MTF for the standard CT algorithm is 5.5 cm^-1^ whereas for the DynaCT images it ranges from 4.8 cm^-1^ to 4.3 cm^-1^. The apparent dependence of MTF on mAs is due to uncertainty in the measurement method. Images with a low signal to noise ratio will produce MTF curves with more statistical variation and hence will exhibit some difference in MTF values, especially at lower modulations.

**Table 5:** Modulation Transfer Function using standard reconstruction parameters

**Dose Level (μGy/frame)**	**Rotation Time (s)**	**kV**	**Average mA**	**Image Slice Thickness (mm)**	**MTF 50% (lpcm^-1^)**	**MTF 10% (lpcm^-1^)**
0.36	5	70	370	10	2.5	4.8
0.36	10	70	360	10	2.5	4.6
0.36	20	71	350	10	2.4	4.3
CT Standard	2	120	120	10	3.3	5.5
CT Edge	1	120	150	1.25	8.8	11.9

## CONCLUSION

Clearly DynaCT is inferior to CT in imaging terms and should not be thought of as bringing diagnostic CT scanning capabilities to the interventional suite. Overall, DynaCT offers inferior image quality compared to conventional CT using same phantoms and typical exposure parameters. The low contrast resolution is significantly worse. This is not surprising given the differences in equipment characteristics (matrix flat panel versus ceramic solid state detectors), the reduction in the number of projections acquired and the differing reconstruction techniques and corrections. The surface dose is higher than in conventional CT for lower contrast.

However, although noise and CT number accuracy are inferior to conventional CT, they are probably not significant, given the intended use of the technology. For example, the noise levels of 1-1.5% are lower than those routinely found in abdominal or high resolution head CT investigations [6]. Although subject to some error, CT numbers are of the right order. High contrast spatial resolution is worse but not significant compared to conventional CT. These latter characteristics render DynaCT suitable for at least some clinical applications proposed, such as detection of brain haemorrhages or identification of abdominal pathology (such as assessment of the outcome of tumour embolisation immediately following the procedure) where low contrast visualisation is not a dominant factor. The advantage is that these 3D imaging procedures, which do not require the exceptional low contrast discrimination provided by conventional CT scanners, can be performed in the interventional suite when required and will remove the necessity for an ‘intra procedure’ CT scan. Thus if DynaCT (or its equivalents) is employed as an adjunct to the imaging acquisition presently used in the vascular suite; it has the potential and capability, at least in imaging terms, to be a valuable tool in the interventional environment.
